# 基于微滴式数字PCR建立血管免疫母细胞性T细胞淋巴瘤IDH2基因突变检测方法

**DOI:** 10.3760/cma.j.cn121090-20241016-00396

**Published:** 2025-06

**Authors:** 茜 黄, 祎 缪, 晓 肖, 静 李, 晖 金, 建勇 李, 文瑜 施

**Affiliations:** 1 南通大学附属医院肿瘤科、血液科，南通 226001 Department of Oncology, Department of Hematology, Affiliated Hospital of Nantong University, Nantong 226001, China; 2 南通大学医学院，南通 226001 Medical School of Nantong University, Nantong 226001, China; 3 南京医科大学第一附属医院、江苏省人民医院血液科淋巴瘤中心，南京 210029 Lymphoma Center, Department of Hematology, Jiangsu Province Hospital, The First Affiliated Hospital of Nanjing Medical University, Nanjing 210029, China; 4 上海源奇生物医药科技有限公司，上海 200001 Shanghai Yuanqi Biomedical Technology Co., Ltd, Shanghai 200001, China; 5 长春理工大学物理学院，长春 130022 School of Physics, Changchun University of Science and Technology, Changchun 130022, China

**Keywords:** 淋巴瘤，T细胞，外周, 淋巴瘤，T细胞，血管免疫母细胞性, IDH2, 突变检测, 微滴式数字PCR, Lymphoma, T-cell, peripheral, Lymphoma, T-cell, angioimmunoblastic, IDH2, Mutation detection, Droplet digital PCR

## Abstract

**目的:**

探究基于微滴式数字PCR（droplet digital PCR，ddPCR）技术建立血管免疫母细胞性T细胞淋巴瘤（angioimmunoblastic T-cell lymphoma，AITL）循环游离DNA（cell-free DNA，cfDNA）中IDH2基因突变的检测方法，并对该方法进行性能评估。

**方法:**

针对IDH2 p.R172K、IDH2 p.R172M和IDH2 p.R172W突变，设计特异性引物探针，优化不同引物组合，建立ddPCR最佳反应体系并通过来源于南京医科大学第一附属医院40例AITL患者的临床cfDNA样本评估该方法的准确度、精密度、分析特异性及检出限。

**结果:**

通过优化引物组合，选用IDH2 R172K/M/W F2/R2引物组合建立IDH2 R172K/M/W突变的ddPCR检测方法。采用特异性标准品检测未观察到假阳性事件；准确度标准品测量结果相对偏差均在±10％范围内；检出限测试表明，所建立的方法能够检出浓度为1 ng/µl、突变比率为0.2％的IDH2 R172K/M/W突变；不同实验者、轮次、实验室的精密度测量结果变异系数均<5％；对40例IDH2 R172K/M/W突变型的临床样本进行基因检测，结果与基因测序结果完全一致。

**结论:**

ddPCR可检测AITL的cfDNA中IDH2基因的突变状态和丰度，具有操作简便、快速、灵敏度和特异性强的优点，能够实现绝对定量，可应用于AITL的诊断和病情监测。

## Abstract (en)

**Objective:**

To establish a method to detect IDH2 gene mutations in the cell-free DNA（cfDNA）sample of angioimmunoblastic T-cell lymphoma（AITL）based on the digital droplet PCR（ddPCR）method. The performance of this method was evaluated.

**Methods:**

Specific primers and probes were designed for IDH2 p. R172K, IDH2 p. R172M, and IDH2 p. R172W mutations, and primer combinations were optimized to establish the optimal ddPCR reaction system. In addition, the accuracy, precision, specificity, and detection limit of the method were evaluated using clinical cfDNA samples obtained from 40 patients with AITL at the First Affiliated Hospital of Nanjing Medical University.

**Results:**

The IDH2 R172K/M/W F2/R2 primer combination was selected to establish the ddPCR detection method for IDH2 R172K/M/W mutations. Specific standard samples showed no false-positive results. The accuracy test revealed that the relative deviation of the standard sample measurements was within ±10％. The detection limit test indicated that the method could detect 1 ng/µl with a 0.2％ mutation rate for IDH2 R172K/M/W mutations. The precision measurements（inter-experimenter, inter-run, and inter-laboratory）all had a coefficient of variation of <5％. ddPCR was applied to 40 clinical samples with IDH2 R172K/M/W mutations, and the results were fully consistent with gene sequencing.

**Conclusion:**

ddPCR can detect IDH2 gene mutations in cfDNA from AITL with high sensitivity, specificity, and absolute quantification. This method offers a simple, rapid, and reliable approach for diagnosing and monitoring.

血管免疫母细胞性T细胞淋巴瘤（angioimmunoblastic T-cell lymphoma，AITL）是外周T细胞淋巴瘤（peripheral T-cell lymphoma，PTCL）最常见的亚型。AITL具有高度异质性和侵袭性，其临床表现和病理特征复杂多样，诊断难度大，易被误诊[1]–[6]。约68％的AITL患者确诊时已是晚期（Ⅲ～Ⅳ期），5年无进展生存率仅为30％，且缓解后复发率高[7]。因此，开发新的诊断技术与分子标志物对于AITL诊断试剂盒的研制、治疗策略的优化以及改善患者预后具有重要的临床意义。

研究显示，AITL肿瘤细胞的发生与TET2、DNMT3A、IDH2和RHOA等基因在造血干细胞/祖细胞中发生表观遗传突变有关，这些突变导致细胞内遗传表观修饰异常和信号通路紊乱[8]。近年来，二代测序（NGS）结果显示，20％～30％的AITL患者携带IDH2 R172突变[9]，而外周T细胞淋巴瘤-非特指型（PTCL-NOS）及其他PTCL亚型中较少检测到该突变。因此，IDH2基因突变在AITL中的高频和特异性分布，凸显其在PTCL亚型的诊断及鉴别中的重要价值。

微滴式数字PCR（droplet digital PCR，ddPCR）技术是一种绝对定量PCR检测方法。其原理是将反应体系分散到数万个油包水的微滴中，在每个微滴中独立进行PCR扩增及荧光信号检测，最后通过泊松分布原理计算出野生型和突变型的初始拷贝数[10]–[11]。近年来，ddPCR凭借样本需求量少、绝对定量能力强、准确度高、分辨率和灵敏度高等优势，在循环游离DNA（cell-free DNA，cfDNA）样本的检测分析中得到了广泛应用。因此，本研究旨在利用ddPCR技术对AITL中IDH2基因的R172K/M/W突变进行方法学开发，为AITL的诊断、鉴别诊断及微小残留病（MRD）的监测提供新的策略，并在患者用药指导及疗效评估方面为临床医师提供支持。

## 对象与方法

一、研究对象

本研究选取来源于南京医科大学第一附属医院40例AITL患者的临床cfDNA样本用于IDH2基因突变检测反应体系的建立与性能评估，其中13例样本为IDH2 R172K/M/W突变阳性、27例为IDH2 R172K/M/W突变阴性。所有40例临床cfDNA样本均经过NGS和ddPCR验证，具体的IDH2基因突变信息详见表1。研究经南京医科大学第一附属医院伦理委员会批准（批件号：2024-SR-463）。

**表1:** 40例血管免疫母细胞性T细胞淋巴瘤患者的临床循环游离DNA样本中IDH2基因突变信息

样本号	IDH2基因突变情况	NGS检测突变丰度（％）	ddPCR检测突变比率（％）
1	IDH2:NM_002168.3:exon4:c.516G>C:p.R172S	7.15	7.76
2	野生型	/	0
3	野生型	/	0
4	IDH2:NM_002168.3:exon4:c.515G>A:p.R172K	2.81	3.37
5	野生型	/	0
6	IDH2:NM_002168.3:exon4:c.514A>G:p.R172G	1.62	1.91
7	野生型	/	0
8	IDH2:NM_002168.3:exon4:c.516G>T:p.R172S	1.49	1.79
9	IDH2:NM_002168.3:exon4:c.516G>T:p.R172S	/	0
10	IDH2:NM_002168.3:exon4:c.515G>A:p.R172K	4.57	5.05
11	野生型	/	0
12	野生型	/	0
13	野生型	/	0
14	IDH2:NM_002168.3:exon4:c.515G>A:p.R172K	13.95	14.80
15	野生型	/	0
16	IDH2:NM_002168.3:exon4:c.515G>A:p.R172K	1.16	1.42
17	IDH2:NM_002168.3:exon4:c.514A>G:p.R172G	12.10	12.50
18	IDH2:NM_002168.3:exon4:c.515G>A:p.R172K	5.82	6.38
19	野生型	/	0
20	野生型	/	0
21	IDH2:NM_002168.3:exon4:c.514A>G:p.R172G	8.86	9.43
22	野生型	/	0
23	IDH2:NM_002168.3:exon4:c.515G>T:p.R172M	2.73	3.17
24	IDH2:NM_002168.3:exon4:c.514A>G:p.R172G	1.36	1.56
25	野生型	/	0
26	IDH2:NM_002168.3:exon4:c.515G>T:p.R172M	9.79	10.50
27	IDH2:NM_002168.3:exon4:c.515G>A:p.R172K	18.77	19.60
28	野生型	/	0
29	IDH2:NM_002168.3:exon4:c.515G>A:p.R172K	3.44	3.75
30	野生型	/	0
31	野生型	/	0
32	IDH2:NM_002168.3:exon4:c.514A>T:p.R172W	9.77	10.20
33	野生型	/	0
34	野生型	/	0
35	IDH2:NM_002168.3:exon4:c.516G>C:p.R172S	2.14	2.56
36	IDH2:NM_002168.3:exon4:c.514A>G:p.R172G	19.13	20.10
37	野生型	/	0
38	IDH2:NM_002168.3:exon4:c.515G>A:p.R172K	4.09	4.32
39	IDH2:NM_002168.3:exon4:c.515G>A:p.R172K	9.82	10.70
40	IDH2:NM_002168.3:exon4:c.514A>T:p.R172W	23.84	24.70

**注** NGS：二代测序；ddPCR：微滴式数字PCR；/：NGS未检出

二、标准品的制备

本研究采用的野生和突变标准品为上海生工生物工程有限公司采用基因重组技术将目的片段序列克隆入pMD-18T载体中，最终通过连接、重组、转化和测序鉴定得到对应IDH2 R172K突变型、R172M突变型、R172W突变型和IDH2 R172野生型的质粒。

1. IDH2标准品的制备：将IDH2在同浓度下的3个突变型质粒及野生型质粒按1∶1∶1∶7混合成10％突变比率的标准品，并用该标准品再与野生型质粒1∶9混合得到1％突变比率的标准品，以此类推得到0.2％、0.02％突变比率的标准品，配置的标准品用于IDH2基因突变检测反应体系的建立与优化。

2. 特异性和准确度标准品的制备：将18例NGS检测结果为阴性的临床样本，与非IDH2 R172K/M/W（样本35）突变型样本用于特异性评估；将已知NGS测序突变比率的IDH2 R172K/M/W突变阳性临床样本（样本39、样本26和样本32）用于准确度评估。

3. 检出限标准品的制备：分别将IDH2 R172K/M/W突变阳性临床样本（样本39、样本26和样本32），按照NGS测序得到的突变比率，分别与等浓度（2 ng/µl）的阴性临床样本（样本19）梯度稀释为10％、5％、1％、0.2％、0.1％、0.05％六个突变比率，再分别将制备的每个突变比率的样本浓度梯度稀释为1 ng/µl和0.5 ng/µl，将配置得到的3个不同量的6个不同突变比率的突变型样本用于灵敏度评估。

4. 精密度标准品的制备：将IDH2在同浓度下的3个突变型质粒和野生型质粒按1∶1∶1∶7混合成10％突变比率的高精密度标准品（IDH2-J），并用该标准品再与野生型质粒1∶9混合得到1％突变比率的中精密度标准品（IDH2-LJ），并用该标准品再与野生型质粒1∶4混合得到0.2％突变比率的低精密度标准品（IDH2-HLJ），将所有配置得到的标准品用于不同实验者、轮次及实验室间精密度评估。

三、探针引物设计

PubMed网站（https://pubmed.ncbi.nlm.nih.gov/）上查询IDH2原始序列，并结合COSMIC网站（https://cancer.sanger.ac.uk/cosmic）查找对应突变位点信息。采用小沟结合物（MGB）探针法设计探针；利用Primer Premier 5.0软件，对IDH2 p.R172K（c.515G>A）、IDH2 p.R172M（c.515G>T）和IDH2 p.R172W（c.514A>T）位点进行引物和探针的设计。所有引物和探针均由上海生工生物工程有限公司合成。按配制需求将引物探针稀释到50 µmol/L，采用设计的引物探针通过自制的标准品进行筛选后确立IDH2对应的反应体系。引物探针序列见表2。

**表2:** IDH2 R172突变引物探针序列

特异性引物探针	核苷酸序列及荧光标记
F1	5ʹ-AGCCCATCATCTGCAAAAAC-3ʹ
R1	5ʹ-CCTTGTACTGCAGAGACAAGA-3ʹ
F2	5ʹ-CGGGAGCCCATCATCTGC-3ʹ
R2	5ʹ-GTGCCCAGGTCAGTGGATC-3ʹ
P-WT	VIC-5′CACCATTGGCAGGCACGC-3′ MGB
P-R172K	FAM-5′CACCATTGGCAaGCACGC-3′ MGB
P-R172M	FAM-5′CACCATTGGCAtGCACGC-3′ MGB
P-R172W	FAM-5′CACCATTGGCtGGCACGC-3′ MGB

**注** F为正向引物；R为反向引物；P为小沟结合物（MGB）探针

四、ddPCR反应体系的建立与优化

以质粒配制的不同突变比率的标准品作为对应反应液的阳性检测样本；以野生型质粒作为对应反应液的阴性检测样本；以工艺用水作为空白对照，建立并优化IDH2 ddPCR反应体系。反应体系总体积为20 µl，包括阳性对照品、阴性对照品、空白对照品各6 µl，350 nmol/L的PCR上下游引物（F1/R1、F2/R2）各0.15 µl，180 nmol/L的野生型和突变型的MGB探针各0.05 µl以及2X的super mix 10 µl。反应体系不足20 µl用无酶水补齐。将配置好的ddPCR反应液移至DG8 Cartridges for QX200 Droplet Generator（美国Bio-rad公司），加入70 µl Generation oil（美国Bio-rad公司），安装Droplet Generator DG8 Gasket（美国Bio-rad公司），将其置于Bio-Rad QX200微滴生成仪（美国Bio-rad公司）内完成微滴的制备。将制备好的微滴转移至96孔板对应的反应孔，铝膜热封96孔板（180 °C，10 s），置于Agilent SureCycler 8800 PCR仪（美国Agilent公司）上进行扩增，设定反应条件：95 °C预变性10 min；94 °C，15 s，58 °C，60 s，共40个循环；98 °C，10 min；4 °C，5 min，反应体系设为40 µl，注意升降温速度≤2 °C/s。将扩增后的96孔板放入QX200微滴读取仪（美国Bio-rad公司），荧光信号选择“（FAM/VIC）”，进行微滴读取及荧光信号检测。检测完成后，点击“2D Amplitude”查看通道1和通道2聚类图。此图允许手工或自动调节阈值对每个检测通道进行阳性和阴性微滴指定。FAM标志为突变基因，如果该通道检测出荧光信号，说明该检测样本中存在突变基因，VIC标志为野生基因，可作为判断反应体系内核酸分子是否成功扩增，还可评估DNA模板的上样量。应用本研究建立的检测方法检测配置用于分析特异性、准确度、检出限、精密度的标准品，评估该检测方法的分析性能。

五、统计学处理

连续变量以*x*±*s*表示，使用相对偏差和变异系数（CV值）分别对检测方法的准确度和精密度进行统计学分析。相对偏差<10％以及CV值<5％认为有统计学意义。

## 结果

一、IDH2基因突变检测反应体系的建立与优化

本研究采用ddPCR技术建立了IDH2 R172K/M/W突变检测方法。通过对阳性样本、阴性样本及空白对照的扩增结果分析，观察到IDH2 R172K/M/W F2/R2引物混合的反应体系检测阳性标准品突变率的准确度最高，且在阴性样本中表现出较强的特异性，结果见表3，同时IDH2 F2/R2检测数据未发现非特异性突变信号（Ch1+Ch2−点数）。经过不同引物组合的优化，最终确定IDH2 R172K/M/W F2/R2引物混合作为IDH2 R172K/M/W突变检测方法的反应体系。

**表3:** IDH2基因突变检测反应体系优化数字结果

样本名称	Ch1+Ch2−点数 （个）	Ch1−Ch2+点数 （个）	FAM通道拷贝数 （copies/µl）	VIC通道拷贝数 （copies/µl）	微滴总数 （个）	突变比率 （％）
IDH2（10％）	289	2 722	32.2	286	13 074	10.12
IDH2（1％）	31	3 390	3.8	371	12 555	1.00
IDH2（0.2％）	4	3 893	0.8	373	14 328	0.22
IDH2（0.02％）	0	2 495	0	266	12 340	0
negative	0	2 911	0	224	16 806	0
H_2_O	0	0	0	0	8 366	0

**注** Ch1+Ch2−点数：FAM（突变型）检测通道的点数；Ch1−Ch2+点数：VIC（野生型）检测通道的点数；negative：阴性对照；H_2_O：水，为空白对照

二、IDH2 R172K/M/W突变检测方法的性能评估

1. 特异性：将NGS检测结果为IDH2突变阴性的18例临床样本分别进行IDH2基因突变检测，在不同野生型靶标拷贝数条件下，均未观察到假阳性事件，突变比率［突变型拷贝数/（突变型拷贝数+野生型拷贝数）］均为0。此外，选取非IDH2 R172K/M/W突变型的临床样本（样本35）的cfDNA进行IDH2基因突变检测，结果未检测出相应突变，突变比率同样为0。

2. 准确度：采用10％突变比率的IDH2 R172K/M/W突变的标准品进行3次重复测定，其中IDH2 R172K突变比率为（10.47±0.12）％，CV值为1.10％；IDH2 R172M突变比率为（10.53±0.12）％，CV值为1.10％；IDH2 R172W突变比率为（10.47±0.15）％，CV值为1.46％。测量结果相对偏差均在±10％范围内，表明本研究建立的检测方法具有较好的准确度。

3. 检出限的建立：对不同浓度下6种不同突变比率的突变型模板进行3次重复的IDH2基因突变检测，1 ng/µl、0.2％突变比率样本3次重复的阳性突变点数（Ch1+Ch2−点数）分别为13、12、9，均检出为阳性；1 ng/µl、0.1％突变比率样本3次结果的阳性突变点数（Ch1+Ch2−点数）分别为2、2、1，检出为可疑阴性。由此初步确定IDH2基因突变检测方法的检出限为能够检出1 ng/µl、0.2％的IDH2突变。

4. 检出限的验证：根据确定的试剂检测限，将IDH2突变型的DNA样本（样本23、样本29、样本40）与等浓度（1 ng/µl）的阴性临床样本（样本20）梯度稀释为0.2％、0.1％，分别进行20次重复检测。结果显示试剂盒检测阴性样本均未检测到突变且FAM检测通道（Ch1+Ch2−）的点数均为0。试剂盒检测1 ng/µl 0.1％突变的IDH2阳性样本的阳性检出率为75％～80％，检测1 ng/µl 0.2％突变的IDH2阳性样本的阳性检出率为95％～100％，且阳性样本FAM检测通道（Ch1+Ch2−）的点数均≥4。因此，确定该试剂盒检出限：1 ng/µl的浓度下突变比率为0.2％且FAM检测通道（Ch1+Ch2−）的点数≥4的IDH2突变。

5. 精密度：对配置得到的高精密度标准品IDH2-J（10％）、中精密度标准品IDH2-LJ（1％）和低精密度标准品IDH2-HLJ（0.2％）分别进行实验者间（A、B、C）、轮次间（1、2、3）及实验室间（a、b、c）精密度评估。根据表4检测结果计算得到的实验者间（A、B、C）、轮次间（1、2、3）及实验室间（a、b、c）精密度CV值均<5％，该结果表明本研究建立的检测方法具有较好的重复性。

**表4:** IDH2精密度检测结果

样本	实验者间精密度（％）		轮次间精密度（％）		实验室间精密度（％）	
	均值±标准差	变异系数	均值±标准差	变异系数	均值±标准差	变异系数
高精密度标准品（J）	10.32±0.43	4.15	10.51±0.28	2.71	10.13±0.28	2.77
中精密度标准品（LJ）	1.06±0.05	4.91	1.08±0.04	3.55	1.12±0.04	3.93
低精密度标准品（HLJ）	0.23±0.01	3.12	0.23±0.01	4.11	0.22±0.01	4.93

## 讨论

本研究首次提出ddPCR能高精准检测出AITL患者的IDH2基因突变，且自主设计了IDH1/2突变特异性探针引物。在设计方法中，我们采用MGB法设计探针，该探针在检测的特异性上优于TaqMan探针[12]，并将突变位点置于探针设计的前中端以降低错配的概率。通过实验结果可以看出本研究建立的ddPCR检测方法具有良好的特异性、灵敏度、准确度以及精密度。该方法在1 ng/ml的浓度下检测IDH2突变可达到0.2％的灵敏度，检测高、中、低精密度标准品CV值均<5％。ddPCR可以将单个靶分子划分在不同的区室中，能够以高精密度和灵敏度检测罕见序列。与传统的实时荧光定量PCR相比，ddPCR的绝对定量不依赖于校准曲线，从而克服了传统方法的局限性[13]。尽管ddPCR无法进行大规模并行检测或发现新的未知突变，但在已知目标突变的检测中，ddPCR较NGS具有明显的经济和时间优势。NGS不是定量方法，其依赖于阈值和定量循环（Cq）值来解释结果，而ddPCR与Cq值无关，且仅需较少量的初始DNA[14]。基于ddPCR的优良性能，该方法不仅可以作为常规检测技术实现诊断、鉴别诊断及疗效监测，将来还可能用于检测AITL中的MRD，从而抢先治疗，预防或延迟血液学复发。

尽管AITL患者采用了积极的治疗方案（如化疗和造血干细胞移植），但复发仍然是主要问题，早期并准确地检测MRD是近年研究热点。鉴于cfDNA侵入性低、可多次取材的方便性，MRD监测为治疗后临床缓解患者检测疾病的持续或复发提供了精细的风险分层和治疗决策方法。目前，用于MRD检测的多种技术主要是基于免疫学方法的多参数流式细胞术（multiparametric flow cytometry，MFC）和基于分子学方法的PCR或NGS。虽然MFC在检测细胞表面标志物方面具有较高的灵敏度，但在检测低丰度肿瘤细胞时会受到多种因素的干扰，如细胞表面标志物的表达差异、细胞亚群的比例变化等，准确度低于NGS。而NGS对MRD检测的灵敏度随着DNA输入量的增加而增加，因此成本高、耗时长、对样本质量和实验条件要求较高等使其难以广泛应用到临床。ddPCR被认为是第三代PCR，在MRD监测中，它的主要优点是无需使用诊断样品稀释度构建的参考标准曲线，并且可直接、绝对、准确地检测和定量低丰度DNA，在MRD定量方面具有优势[15]。因此，NGS检测大规模基因的能力可以有效检测和确认AITL患者的IDH2突变，从而使AITL患者选择有效的治疗方式和药物。而ddPCR的高灵敏度以及多次取样的优势，能对AITL患者进行有效的MRD监测，两者的结合使用，对于提升AITL患者预后具有重要的临床意义。

IDH2异常表达可干扰细胞代谢，促进肿瘤发生，是AITL的遗传标志物，其诊断滤泡辅助性T细胞表型的AITL和PTCL的价值越来越高。IDH2抑制剂Enasidenib可逆转组蛋白和DNA的过度甲基化，是一种新型有效的、具有选择性的IDH2突变酶的可逆抑制剂[16]–[17]。目前Enasidenib主要用于复发或难治性急性髓系白血病（AML）的治疗，并被用在IDH2基因突变的神经胶质瘤、胆管癌和软骨肉瘤等实体瘤中进行试验研究[18]–[19]。在IDH2基因突变的AITL中Enasidenib表现出一定的治疗潜力。因此，Enasidenib对AITL的治疗潜力有可能使IDH2基因突变的检测在AITL中成为一项新的临床需求。IDH2基因突变与多种肿瘤的发生发展有关，包括AML、胶质母细胞瘤和低级别胶质瘤等。因此，IDH2基因突变的检测对于多种癌症治疗至关重要。一项回顾性研究[20]发现，在AML中IDH2基因突变是MRD监测的可靠标志物。而IDH2基因突变是否可成为AITL以及其他血液恶性肿瘤的MRD监测的标志物，仍需进一步研究与探索。

本研究成功建立了IDH2基因突变的ddPCR检测方法，该方法在1 ng/ml的浓度下即可检测出IDH2突变，检测灵敏度可达0.2％，且在高、中、低精密度标准品中，CV值均小于5％。实验中采用IDH2 R172K/M/W F2/R2引物混合体系，实现了IDH2野生型和突变型的高效检测。通过特异性验证和检出限的评估，该方法显示出良好的特异性和灵敏度。综上所述，本研究建立的ddPCR检测方法可作为MRD、稀有突变或低拷贝靶标检测的有效工具，为携带IDH2突变的AITL患者提供准确的诊断依据，进而辅助临床制定个体化和精准的治疗方案，未来仍需更大样本进一步验证并完善该方法。
